# Incorporation of covariates in simultaneous localization of two linked loci using affected relative pairs

**DOI:** 10.1186/1471-2156-11-67

**Published:** 2010-07-14

**Authors:** Yen-Feng Chiu, Jeng-Min Chiou, Kung-Yee Liang, Chun-Yi Lee

**Affiliations:** 1 Division of Biostatistics and Bioinformatics, Institute of Population Health Sciences, National Health Research Institutes, 35 Keyan Rd., Zhunan, Miaoli 350, Taiwan; 2 Institute of Statistical Science, Academia Sinica, Taipei, Taipei, Taiwan; 3 Department of Biostatistics, Bloomberg School of Public Health, Johns Hopkins University, USA; 4 National Yang-Ming University, Taipei, Taiwan

## Abstract

**Background:**

Many dichotomous traits for complex diseases are often involved more than one locus and/or associated with quantitative biomarkers or environmental factors. Incorporating these quantitative variables into linkage analysis as well as localizing two linked disease loci simultaneously could therefore improve the efficiency in mapping genes. We extended the robust multipoint Identity-by-Descent (IBD) approach with incorporation of covariates developed previously to simultaneously estimate two linked loci using different types of affected relative pairs (ARPs).

**Results:**

We showed that the efficiency was enhanced by incorporating a quantitative covariate parametrically or non-parametrically while localizing two disease loci using ARPs. In addition to its help in identifying factors associated with the disease and in improving the efficiency in estimating disease loci, this extension also allows investigators to account for heterogeneity in risk-ratios for different ARPs. Data released from the collaborative study on the genetics of alcoholism (COGA) for Genetic Analysis Workshop 14 (GAW 14) were used to illustrate the application of this extended method.

**Conclusions:**

The simulation studies and example illustrated that the efficiency in estimating disease loci was demonstratively enhanced by incorporating a quantitative covariate and by using all relative pairs while mapping two linked loci simultaneously.

## Background

With the advance of genotyping techniques, genome-wide association analysis has become the mainstream technique in genetic mapping. However, studies have shown that using information from linkage scans can improve the power of association mapping in genome scans [1]. In addition, linkage analysis could be more powerful than association analysis for some genetic mechanisms; family data can also help to estimate familial risks [2]. Hence, linkage analysis remains a useful and supplemental tool to map genes for complex diseases. As complex diseases often involve quantitative biomarkers or environmental factors, incorporating these quantitative factors into linkage mapping can improve the power to detect disease loci [3] or the efficiency of estimating disease loci. Efficiency is defined as the inverse of the variance estimate for the disease locus estimate. Thus, smaller variance estimates have higher efficiencies. Moreover, the incorporation of covariates provides information that can be used to characterize disease loci, which is important for understanding disease etiologies and mechanisms and for identifying population subgroups that may have particularly high disease risks [4]. Methodologic work has demonstrated that failure to adequately account for gene-covariate interaction in a genetic analysis can mask the effects of both genes and covariates [5-7]. Hence, it is important to develop linkage approaches that allow the inclusion of covariates.

Thus far, several linkage analyses including covariates have been proposed to account for linkage heterogeneity or to examine biological, environmental, gene-gene or gene-environment interaction effects. Devlin (2002) [5] accounts for linkage heterogeneity by incorporating a family-level covariate into likelihood-based mixture models; however, this approach accounts for linkage heterogeneity only. Greenwood and Greenwood (1997, 1999) [6,8] incorporated covariates into genome scanning approaches using sib-pair or relative-pair through model-based logarithms of odds (LOD) score approaches, where the generalized expected identity-by-descent (IBD) sharing was modeled as a function of some covariates through multinomial logistic regression. Rice (1999) [7] applied a novel technique to detect significant covariates in linkage analyses with a logistic regression approach using all sib pairs (concordant affected, concordant unaffected, and discordant), and Saccone et al. (2001) [9] further extended this analysis to cousin pairs. Olson (1999) [10] proposed a unified framework for model-free linkage analysis that can handle the separate inclusion of other ARPs, discordant relative pairs, covariates, or additional disease loci through a conditional-logistic parameterization. These regression-based approaches can easily be generalized to include all covariates; however, they assume either one disease locus or multiple unlinked loci and thus are not applicable to analyses of multiple linked loci. For non-regression-based approaches, Hauser et al. (2004) [11] proposed a model-free LOD scores approach that includes family-level covariate information. This approach also assumes only one disease locus and can only incorporate one covariate at a time. In addition, the problem of multiple testing may arise when researchers perform multiple tests or analyses using various combinations of multiple loci or covariates using these approaches.

On the other hand, most two-locus linkage approaches aim to detect the presence of a second susceptibility gene by accounting for the effects of a known susceptibility gene [12-14]. However, when two susceptibility loci are linked, the location of the first gene may be inaccurate because it was mapped without accounting for the effects of the linked gene. Thus, conditional analyses that rely on an inaccurate position for the first locus may result in an inaccurate second disease loci estimate as well. Biswas et al. (2003) [15] applied a Bayesian approach to simultaneously detect two linked disease genes; however, their approach was designed to detect genes under locus heterogeneity only, and this model-based approach requires the specification of unknown genetic parameters. Hence, linkage approaches that can simultaneously localize two linked disease genes are in great demand.

Rather than testing the presence of linkage, Liang et al. (2001) [16] developed a novel, robust, model-free multipoint linkage method that simultaneously estimates both the position of a disease locus as well as its effect on the disease, along with its sampling uncertainty. The advantages of this method include: (i) It does not require specification of an underlying genetic model; hence, estimation of the parameters is robust to a wide variety of genetic mechanisms. (ii) The multiple testing issue is eliminated as a single test statistic is provided for linkage in the entire studied region; rather than testing the hypothesis for one marker at a time. (iii) While multiple markers are incorporated simultaneously in the gene mapping, there is no need to specify the phase of genotypic data with multiple markers. Many complex diseases, such as hypertension, schizophrenia, diabetes, and asthma are usually defined as dichotomous phenotypic traits; however, they are also associated with quantitative biological markers or quantitative risk factors. As a result, Glidden et al. (2003) [17] further incorporated quantitative covariates into Liang's approach [16] and estimated the genetic effect of a disease locus through a logistic-type parametric model using affected sib pairs (ASPs). Based on the same study design, Chiou et al. (2005) [18] incorporated quantitative covariates into their linkage mapping and estimated the genetic effect of a disease locus non-parametrically. This quantitative covariate could be either an environmental risk factor or itself a quantitative trait. For the quantitative trait incorporated as a covariate, its QTL (quantitative trait locus) may directly underlie a pathway of the disease or be linked to the disease locus, or the trait may be indirectly associated with the disease.

Meanwhile, Schaid et al. (2005) [19] extended the without-a-covariate approach by Liang et al. [16] to different types of ARPs. The authors' extension relaxed the limitation to ASPs only and allowed an investigator to study the risk-ratios of a disease gene estimated from multiple relative pairs; this work helped to uncover the underlying genetic mechanism of disease. To jointly localize two linked disease loci using ASP data, Biernacka et al. (2005) [20] extended this approach [16] to the localization of two linked disease-susceptibility genes. They also provided tests for the presence of two linked disease-susceptibility genes by a quasi-likelihood ratio test and a modified score test in another article [21]. Lin and Schaid (2007) [22] generalized the two-locus localization method to a variety of ARPs. Both of the unconstrained and constrained models, along with a score test and the examination of the goodness of fit of a used constrained model, were described in their generalized method. As the etiology of complex diseases often involves quantitative variables (either genetic biomarkers or environmental factors) in addition to multiple disease loci, it is helpful to incorporate a quantitative variable while localizing two linked disease loci simultaneously using ARPs. We extended Lin and Schaid's (2007) [22] approach to incorporate quantitative covariates in two-locus linkage mapping using ARPs. Generally, a statistical parametric model is simpler and easier to interpret than a non-parametric model, while a non-parametric model has the flexibility to fit the data perfectly. To take advantages of parametric and non-parametric statistical models, we applied both models to incorporate covariates. These methods can also be applied to account for heterogeneity from quantitative covariates as well as from multiple subgroups that are stratified by categorical covariates. Systematic simulation studies under a variety of quantitative covariates were conducted to evaluate the gain in efficiency of estimating the disease loci from the proposed methods. The estimates from the proposed approaches with incorporation of covariates were compared with those from the approach without incorporating covariates. The collaborative study on the genetics of alcoholism (COGA) data released for GAW14 was used to illustrate the proposed approaches.

## Methods

To incorporate relevant covariate information while simultaneously estimate the locations of two genes using all types of relative pairs in linkage analysis, we proposed the following linkage approaches.

### Simultaneous Localization of Two Linked Disease Susceptibility Genes with Incorporation of Covariates

Consider a chromosomal region harboring two linked disease loci, *τ*_1 _and *τ*_2_, with *M *markers genotyped at the locations 0 = *t*_1 _<*t*_2 _< ⋯ <*t*_*M*_. Let *S*_*ki*_(*t*_*j*_) be the identity-by-descent (IBD) sharing for the *j*^th ^marker of the *i*^th ^pair of the ARP type *k*, *j *= 1,...,*M*, *i *= 1,...,*n*_*k*_, *k *= 1,...,5. The five types of relative pairs considered include full siblings (SP, *k *= 1), half siblings (HS, *k *= 2), first cousins (FC, *k *= 3), grandparent-grandchild pairs (GP, *k *= 4) and avuncular pairs (AP, *k *= 5) [19]. The five affected relative pairs are abbreviated as ASP, AHS, AFC, AGP and AAP. Let *x*_*ki*1_, *x*_*ki*2 _be the covariates associated with relatives 1 and 2 in the *i*^*th *^relative pair of type *k*, respectively. Given the covariates and assuming that the recombination fraction does not depend on the covariates, the expectation of IBD sharing at *t*_*j *_for a relative pair *ki *[22] is(1)

where *C*_*lk*_(*x*_*ki*1_, *x*_*ki*2_) = *E*(*S*_*ki *_(*τ*_*1*_)|*x*_*ki*1_, *x*_*ki*2_, Φ)- *a*_*k *_is the genetic effect at locus *l *for a relative pair *ki *;*l *= 1, 2; Φ is the event of an ARP; *d*_1 _= |*τ*_1 _- *t*_*j*_|, *d*_2 _= |*t*_*j *_- *τ*_2_|; *d*_3 _= |*τ*_2 _- *τ*_1_|; *a*_*k *_is the expected count for random sharing; *b*_*k*_(*d*_*v*_) controls the rate of decrease of expected sharing as the distance *d*_*v *_from the trait locus increases; and *v *= 1,2,3. Haldane's mapping function was used to translate recombination fraction to map distance. The values of *b*_*k *_(*d*_*v*_) and *d*_*v *_for each relative type *k *and functions relating the risk ratio *λ *to *C *are listed in supplemental Additional file 1 Table S1 (adopted from Table 1 in Lin and Schaid (2007) [22]).

**Table 1:** Simultaneous two-locus search incorporating quantitative traits with QTLs at *τ*_1_(X_QTL1_) or *τ*_2_(X_QTL2_)

	Disease Loci (cM)				Estimate of C								95% coverage probability (%)				y_*l*_: covariate for modeling C_*l*_, *l *= 1, 2
				
	Parametric		Nonparametric		Parametric				Nonparametric				Parametric		Nonparametric		
								
	*τ*_1_	*τ*_2_	*τ*_1_	*τ*_2_	ASP		AGP		ASP		AGP		*τ*_1_	*τ*_2_	*τ*_1_	*τ*_2_	
													
					C_11_	C_21_	C_14_	C_24_	C_11_	C_21_	C_14_	C_24_					
						
Bias	0.1	-0.1	-0.008	1.1	0.04	0.03	-0.05	0.01	-0.02	-0.05	-0.04	0.02	95	95	93	91	y_1 _= X_QTL1_
Sample variance	4.0	4.0	5.4	6.2	0.003	0.003	0.001	0.001	0.002	0.002	0.001	0.001					y_2 _= X_QTL1_
Mean variance	4.0	4.0	4.8	5.7													
$\overset{\wedge }{\beta_{1}}$					0.26	-0.25	0.16	-0.08									
p-value					0.03	0.05	0.50	0.81									
Bias	0.2	-0.05	-1.1	-0.01	0.04	0.03	-0.05	0.02	-0.04	-0.02	-0.04	0.03	94	95	91	93	Y_1 _= X_QTL2_
Sample variance	4.9	4.2	6.7	5.0	0.003	0.003	0.001	0.001	0.002	0.002	0.001	0.001					y_2 _= X_QTL2_
Mean variance	4.1	3.8	5.9	4.6													
$\overset{\wedge }{\beta_{1}}$					-0.25	0.26	-0.09	0.16									
p-value					0.05	0.03	0.79	0.53									

Bias	0.1	-0.1	-0.5	0.5	0.04	0.03	-0.05	0.02	-0.02	-0.02	-0.04	0.03	94	94	91	91	y_1 _= X_QTL1_
Sample variance	4.5	4.5	5.7	5.4	0.003	0.003	0.001	0.001	0.002	0.002	0.001	0.001					y_2 _= X_QTL2_
Mean variance	3.9	3.8	4.8	4.6													
$\overset{\wedge }{\beta_{1}}$					0.26	0.26	0.16	0.16									
p-value					0.03	0.03	0.50	0.53									

Bias	0.2	-0.1	-0.6	0.6	0.04	0.04	-0.05	0.02	-0.04	-0.05	-0.04	0.02	94	94	91	91	y_1 _= X_QTL2_
Sample variance	5.5	5.6	7.6	6.9	0.003	0.003	0.001	0.001	0.002	0.002	0.001	0.001					y_2 _= X_QTL1_
Mean variance	4.4	4.2	5.9	5.7													
$\overset{\wedge }{\beta_{1}}$					-0.25	-0.25	-0.09	-0.09									
p-value					0.05	0.05	0.79	0.81									

C_1 _and C_2 _represent the amount of excess IBD sharing at each of the two disease gene loci, which is increased by effects due to both disease genes. The simple "effect size" interpretation does not apply to C_1 _and C_2 _in the two-locus model because the magnitude of C_1 _depends not only on the effect of gene 1 but also on the distance between gene 1 and gene 2. C_1 _and C_2 _can each be re-parameterized to represent excess sharing at a location due to the gene at that location and thus can be considered the "effect size" of that particular gene (see Appendix of [20], page 47). They can then be used to test for the presence of linkage. We applied parametric and non-parametric methods to model the association between the excess IBD sharing (*C*_*l*_) at *τ*_*l*_, *l *= 1, 2 and the covariates.

### Parametric Modeling on *C*

In the parametric model, *C*_1*k *_and *C*_2*k *_can be modeled as a function of covariates [17]; an example is the postulation of a logistic regression for IBD sharing at *τ*_1 _and *τ*_2_. For a relative-pair type *k*, assuming *G*_*lk *_= (*g*_*lk*1_,⋯,*g*_*lkp*_)^*T *^is the covariate vector, *C*_1*k *_and *C*_2*k *_were modeled separately, where *g*_*lkr *_= *g*_*lkr*_(*x*_*kr*1_, *x*_*kr*2_), *r *= 1,...,*p*, indicate covariates.

(2) $$C_{lk}(g_{lk})=-\frac{1-\exp(\alpha_{lk}+\beta_{lk}{}^{T}G_{lk})}{f_{k}*(1+\exp(\alpha_{lk}+\beta_{lk}{}^{T}G_{lk}))},$$

where *β*_*lk*_^*T *^= (*β*_*lk*1_,⋯,*β*_*lkp*_), *l *= 1, 2, *k *= 1,...,5; *f*_*k *_= 1 for ASP, *f*_*k *_= 4 for AFC, and, *f*_*k *_= 2 for other ARPs. The gene-environment interaction for environmental variable, *x*_*r*_, could be assessed by examining whether the corresponding *β*-coefficient, *β*_*r*_, is statistically significantly different from zero. In addition, the interactions between two covariates on the genetic effects of the disease loci could also be assessed by adding an interaction term between the two covariates.

### Nonparametric Modeling on *C*

For the non-parametric model, given the data $\left(G_{ki},S_{ki}^{*}({\tilde{\tau }}_{l})\right)$, where *G*_*lki *_= (*g*_*lki*1_,⋯,*g*_*lkip*_)^*T *^with *g*_*lkir*_, = *g*_*lkir*_(*x*_*kir*1_, *x*_*kir*2_), *r *= 1,...,*p*, *i *= 1...,*n*_*k*_, and the imputed IBD sharing $S_{ki}^{*}({\tilde{\tau }}_{l})$ at ${\tilde{\tau }}_{l}$, which is a specified or estimated value of *τ*_*l*_, the estimator of *C*_*lk *_at an arbitrary target *g*_*lk *_= (*g*_*lk*1_,...,*g*_*lkp*_)^*T *^is obtained by ${\overset{\wedge }{C}}_{lk}(g_{lk})={\overset{\wedge }{\beta }}_{lk0}$ such that ${\overset{\wedge }{\beta }}_{lk}=({\overset{\wedge }{\beta }}_{lk0},{\overset{\wedge }{\beta }}_{lk1},...,{\overset{\wedge }{\beta }}_{lkp})$ is the minimizer of the following kernel-weighted least squares function with respect to *β*_*lk *_= (*β*_*lk*0_, *β*_*lk*1_,...,*β*_*lkp*_), ∀*l *= 1, 2,

(3) $$\sum_{k=1}^{5}\sum_{i=1}^{n_{k}}{\left[(S_{ki}^{*}({\tilde{\tau }}_{l})-a_{k})-\beta_{lk0}-\beta_{lk1}(g_{ki1}-g_{k1})-...-\beta_{lkp}(g_{kip}-g_{kp})\right]}^{2}K(H^{-1}(g_{lk}-G_{lki})),$$

where *K *is a p-variate Epanechikov kernel function,

*H *is a nonsingular square bandwidth matrix [18], and *a*_*k *_is the expected count for random sharing [19].

### Estimating *τ*_1 _and *τ*_2_

Given the function *C*_*lk*_(*x*_*ki*1_, *x*_*ki*2_), the trait locus *τ*_*l *_can be estimated by solving the estimating equation [16,18] (4) below. Once the estimate of *C*_*lk *_is obtained, it can be plugged into the equation (4) and the estimate of *τ*_*l *_can be updated. That is, we replace *C*_*lk*_(*x*_*ki*1_, *x*_*ki*2_) with the estimate ${\overset{\wedge }{C}}_{lk}(x_{ki1},x_{ki2})$, which then yields the following estimating equation for *δ *= (*τ*_1_, *τ*_2_):

(4) $${\sum_{k=1}^{5}\sum_{i=1}^{n_{k}}\left(\frac{\partial \mu_{ki}(\delta )}{\partial \delta }\right)}^{\prime }Cov^{-1}(S_{ki})(S_{ki}-\mu_{ki}(\delta ))=0,$$

where *S*_*ki *_= (*S*_*ki*_(*t*_1_),⋯,*S*_*ki*_(*t*_*M*_))', and

$$\mu_{ki}(\delta )=(\mu_{ki}(t_{1};{\overset{\wedge }{C}}_{1k},{\overset{\wedge }{C}}_{2k},\delta ),\cdots ,\mu_{ki}(t_{M};{\overset{\wedge }{C}}_{1k},{\overset{\wedge }{C}}_{2k},\delta )),$$

with $\mu_{ki}(t_{j};{\overset{\wedge }{C}}_{1k},{\overset{\wedge }{C}}_{2k},\delta )=E(S_{ki}(t_{j})|{\overset{\wedge }{C}}_{1k},{\overset{\wedge }{C}}_{2k})$.

The estimates of *C*_*lk *_and *δ *were iteratively updated until the convergent criteria for *δ *were met. Assuming all relative pairs share a common *δ*, the estimates of *δ *follows asymptotic normality (see Additional file 2, Appendix for details) with a mean vector *δ *and a covariance matrix ∑^-1^, where.

$$\Sigma =\sum_{k=1}^{5}\sum_{i=1}^{n_{k}}{\left(\partial \mu_{ki}(\delta )/\partial \delta \right)}^{\prime }Cov^{-1}(S_{ki})(\partial \mu_{ki}(\delta )/\partial \delta )$$

## Simulation Studies

Families with three generations including eight members were simulated: The first generation (4 grandparents) included one or zero affected subjects, the second generation had no affected members, and the third generation included two affected individuals. In total, 200 independent families were simulated, each including one affected sibpair. Of the 200 families, 100 included two affected grandparent-grandchild pairs, with the others not having any affected grandparent-grandchild pairs. Hence, there were 200 ASPs and 200 AGPs per replicate. In total, 1,000 replicates were simulated for each configuration.

### One disease locus model

First, we extended the one-locus model proposed by Schaid et al. (2005) [19] with ARP to incorporate covariates using both parametric modeling [17] and non-parametric modeling [18]. We studied the enhancement of efficiency incurred by the incorporation of a quantitative covariate and by the usage of relative pairs in place of using sib pairs alone within a one-locus model. Three sets of penetrance rates (f_2_, f_1_, f_0_) for the genotypes of two high-risk alleles (f_2_), one high and one low-risk alleles (f_1_), and two low-risk alleles (f_0_) at the disease locus used in the simulation study were (i) (0.67,0.05,0.007) (recessive model), (ii) (0.67,0.55,0.007) (dominant model) and (iii) (0.8,0.4,0.0) (additive model), respectively.

A covariate might be directly or indirectly associated with the disease loci, and the information from covariates under different genetic mechanisms may differentially enhance the search for the disease loci. We studied a variety of covariates correlated with the disease trait under different scenarios: (1) a quantitative trait with a pleiotropic effect (that is to say a quantitative trait that is controlled by the disease locus, *τ*_1_, namely, its QTL is *τ*_1_, yet is not directly associated with liability of the disease); (2) a quantitative trait with a co-incidence effect in which the QTL is linked to a disease locus by incidence, yet does not share common genetic components from the disease locus; (3) a quantitative trait unlinked to the disease loci; (4) a covariate of age at onset with the distribution log*T *= -log *λ*- *βZ *+ *ε*/*γ*, where *Z *is the number of copies of the disease allele [17] at one disease locus. The variable *ε *is distributed as a standard extreme-value random variable with *λ *= 0.03, *γ *= 5.0, and *β *= 0.57; this distribution was built while assuming that the disease allele frequency is 0.05. The distribution of age at onset (T) followed a Weibull distribution, and the disease allele accelerated the onset of disease by a factor of 1.78. The threshold of age at onset was 70.

The quantitative trait **y **for scenarios (1) - (3) follows a multivariate normal distribution ***y***_i _= **μ**_i _+ ***g***_i _+ ***e***_i_, ***e***_i _~ N(**0, Σ**_i_), i = 1,...,*n*, where $y_{i}={\left(y_{1i},y_{2i},...,y_{n_{i}i}\right)}^{T},g_{i}={\left(g_{1i},g_{2i},...,g_{n_{i}i}\right)}^{T}\;\text{and}\;e_{\text{i}}={\left(e_{1i},e_{2i},...,e_{n_{i}i}\right)}^{T}$. *n*_*i *_is the total family members in the *i*^th ^family; **μ **is a *n*_*i *_× l zero vector.

$\Sigma_{i}={\left[\begin{array}{cccc} 0.8 & 0.16 & \cdots & 0.16\\ 0.16 & 0.8 & 0.16 & \vdots \\ \vdots & \vdots & \ddots & 0.16\\ 0.16 & \cdots & 0.16 & 0.8 \end{array}\right]}_{n_{i}\times n_{i}}$; and ***g***_*i *_is a vector of genotypic effects of the QTL. The genotypic effects are 2, 0 and -2 for the genotypes of two high-risk alleles, one high-risk together with one low-risk allele and two low-risk alleles, respectively.

### Two disease locus model

Furthermore, we simulated a two-locus disease model and compared the estimates of *τ*_1 _and *τ*_2 _from approaches with and without incorporating a covariate. We generated the two-locus models of model B in Biernacka et al. [20] as described in Additional file 3, Table S2 to study the impact of covariates on the estimates from the without-a-covariate approach and parametric and non-parametric with-a-covariate approaches.

For genotype data, we generated ten markers that were equally spaced at 10 cM between adjacent markers, with each marker having eight equal-frequency alleles, and the two diallelic disease loci were located at 35 and 75 cM. For scenarios (1), (2) and (3), an additive genetic model for the quantitative trait covariate was assumed. The covariate used in modeling *C*_*l *_was denoted by *y*_*l *_, with *l *= 1,2. Assuming the quantitative traits X_QTL1 _and X_QTL2 _were controlled by *τ*_1_, *τ*_2 _respectively, we examined the impact of different combinations of traits incorporated in functions of *g*_*lk *_on estimating the two trait loci. As in the simulation for the one-locus model, four scenarios were considered for the QTL of each covariate: (1) The QTL is at 35 cM (*τ*_1_) (pleiotropic effect); (2) the QTL for "age at onset" (covariate) is at 35 cM (*τ*_1_); (3) the quantitative trait's QTL is at 45 cM (coincident effect); (4) the covariate's QTL is not linked to either disease locus. All covariates were determined by averaging the two individuals' covariate values in one pair, that is, *g*_*ki*_= (*x*_*ki*1_+ *x*_*ki*2_)/2.

## Results

**Figure 1:**
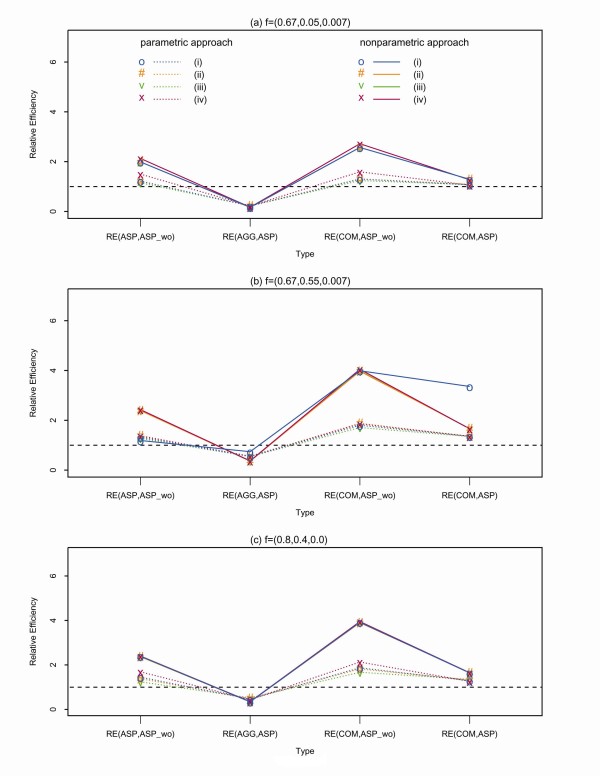
**Relative efficiency (RE) between two approaches in estimating the disease locus under three genetic models (a), (b) and (c)**. The dotted lines are the RE for comparisons between two types of affected relative pairs in the non-parametric approach. The solid lines are the RE for comparisons between two types of affected relative pairs in the parametric approach. ASP, AGG and COM stand for affected sib pairs, affected grandparent-grandchild, and combined affected sib pairs and grandparent-grandchild pairs, respectively. ASP_wo stands for using ASP without incorporating a covariate. The circle, pund, v and x signs refer to the relationship between the covariate's QTL and the disease locus, including (i) pleiotropy, (ii) co-incident, (iii) unlinked, and (iv) a covariate of age at onset.

For the comparison under one-locus models (Figure 1, Additional file 4, Tables S3 - S5), the efficiency in estimating the disease locus was enhanced substantially when incorporating a quantitative covariate, regardless of its underlying genetic mechanisms. In the additive model using affected sibpairs, the relative efficiency (RE) ranged from 1.24 to 1.69 for the parametric approach and from 2.37 to 2.40 for the non-parametric approach. After adding affected grandparent-grandchild pairs, the RE ranged increase to 3.9-3.95 for the parametric approach and 1.67-2.13 for the non-parametric approach. The parametric approach generally had higher RE than the non-parametric approach in the simulated scenarios (Additional file 4, Tables S3 - S5). Given the same heritability of a quantitative trait, incorporating a quantitative trait with a pleiotropic effect was generally more efficient than when incorporating a linked or an unlinked trait. The variance estimate for $\overset{\wedge }{\tau }$ in the one-locus models was generally smaller in the parametric approach than that found in the non-parametric approach under the same scenarios. As expected, with the same sample size, the efficiency in estimating the disease locus was always higher when using affected sibpairs than when using grandparent-grandchild pairs. The efficiency in estimating the disease locus was always improved when combining both relative pairs. The 95% coverage probabilities for the disease locus were almost always slightly underestimated, as most of the variance estimates tended to be underestimated.

The smoothing parameter in (3) was set to one half of the range of the covariates, which roughly minimizes the variance estimate of the estimated loci in the analysis. The choice of bandwidth in the non-parametric approach did not have much impact on the estimation though [18]. The selection of function *g*(·) might slightly influence bias and variance of the estimates for disease loci (these results not shown here). Results from both parametric and non-parametric approaches suggested that the efficiency in estimating disease locus was improved when combining affected sib pairs and grandparent-grandchild pairs.

Since there were two linked loci controlling the disease, we generated covariates X_QTL1 _and X_QTL2_, controlled by *τ*_1 _and *τ*_2_, respectively, and studied the impact of four different ways to incorporate X_QTL1 _or X_QTL2 _into the linkage mapping: (i) incorporating X_QTL1 _only (*y*_1 _= *X*_*QTL*1_, *y*_2 _= *X*_*QTL*1_); (ii) incorporating *X*_*QTL*2 _only (*y*_1 _= *X*_*QTL*2_, *y*_2 _= *X*_*QTL*2_); (iii) incorporating *y*_1 _= *X*_*QTL*1_, *y*_2 _= *X*_*QTL*2 _to estimate *C*_1_, *C*_2_, respectively; (iv) incorporating *y*_1 _= *X*_*QTL*2_, *y*_2 _= *X*_*QTL*1_, to estimate *C*_1_, *C*_2_, respectively. Tables 1 illustrates the impact of choosing different covariates on estimates by parametric and non-parametric approaches, respectively. In reality, we do not have information about the underlying genetic mechanism of the quantitative traits (covariates); luckily, the efficiency in estimating the disease loci was improved under any one of the above scenarios when compared to the estimates made without covariates. Since the quantitative traits were controlled by the two disease loci, incorporating both quantitative traits was helpful in estimating both loci and their 95% coverage probabilities. When incorporating only one quantitative trait, the bias and variance estimate for its corresponding disease locus (QTL) were smaller; this finding was particularly true within the parametric approach. Additionally, both of the covariates were significantly associated with the genetic effects from the two disease loci in the parametric approach (p-values = 0.029 ~ 0.050).

We also evaluated the performance of the parametric and non-parametric approaches with varying locations for covariates' QTLs (Table 2). In the parametric approach, the efficiency in estimating a disease locus was improved when the set location of the covariate's QTL was linked to the disease locus, particularly when the disease locus was also the QTL of the covariate. For example, when no covariate was incorporated, the variance estimates were 7.5 and 6.9 for the two disease loci, respectively (Additional file 5, Table S4); when a quantitative trait with a pleiotropic effect was incorporated, the variance estimates were 4.0 and 4.0 respectively (Table 2). Compared with the estimate without incorporating a covariate, the bias was slightly higher than when the covariate's locus was not the disease locus but was instead linked or unlinked to the disease locus. The biases for estimating the two loci were -0.02 and -0.2 with the pleiotropic covariate and 0.3 and -0.4 with the unlinked covariate (Table 2). In the parametric approach, the magnitude of the regression coefficient reflects the association between the disease locus and the covariate. The regression coefficient was significant only when the covariate's QTL was one of the disease loci (pleiotropy effect) (Table 2). After incorporating a covariate, the 95% coverage probabilities for *τ*_1 _and *τ*_2 _were both more precise than those obtained without incorporating a covariate (Tables 1 and 2; Additional file 5, Table S6). In the non-parametric approach, the efficiency in estimating both disease loci was improved when the covariate's QTL was at position *τ*_1 _(Table 2; pleiotropic covariate or age at onset). The efficiency was lower when the covariate's QTL was linked or unlinked to position *τ*_1 _(Tables 2). The bias was generally higher for *τ*_2 _in the scenario where the covariate provides information for *τ*_1 _only (Tables 2).

**Table 2:** The impact of the location of the QTL for the covariate - parametric and nonparametric approaches

	Disease Loci (cM)				Estimate of C								95% coverage probability (%)				The Location of the Covariate's QTL
				
	Parametric		Nonparametric		Parametric				Nonparametric				Parametric		Nonparametric		
								
	*τ*_1_	*τ*_2_	*τ*_1_	*τ*_2_	ASP		AGP		ASP		AGP		*τ*_1_	*τ*_2_	*τ*_1_	*τ*_2_	
													
					C_11_	C_21_	C_14_	C_24_	C_11_	C_21_	C_14_	C_24_					
											
Bias	-0.02	-0.2	-0.1	1.0	0.04	0.03	-0.05	0.02	-0.02	-0.04	-0.04	0.02	95	96	96	93	
Sample variance	4.4	3.7	4.7	5.9	0.003	0.003	0.001	0.001	0.002	0.002	0.001	0.001					
Mean variance	4.0	4.0	4.8	5.6													
$\overset{\wedge }{\beta_{1}}$					0.26	-0.25	0.16	-0.08									
p-value					0.03	0.05	0.52	0.82									
Bias	0.2	-0.03	0.4	1.8	0.03	0.03	-0.05	0.02	-0.007	-0.06	-0.03	0.02	95	96	93	88	Age onset at 35 cM (*τ*_1_)
Sample variance	4.4	3.9	5.2	5.9	0.003	0.003	0.001	0.001	0.002	0.002	0.001	0.001					
Mean variance	4.1	4.1	4.4	6.0													
$\overset{\wedge }{\beta_{1}}$					-0.04	0.04	-0.03	0.01									
p-value					0.05	0.06	0.54	0.83									

Bias	0.3	-0.3	-0.1	0.7	0.06	0.05	-0.05	0.02	-0.03	-0.04	-0.05	0.02	95	97	95	95	Co-incident 45 cM
Sample variance	6.8	5.7	9.1	8.5	0.003	0.003	0.001	0.001	0.002	0.002	0.001	0.001					
Mean variance	6.7	6.3	8.9	9.0													
$\overset{\wedge }{\beta_{1}}$					-0.007	0.003	-0.002	0.006									
p-value					0.96	0.95	0.94	0.95									

Bias	0.3	-0.4	-0.8	0.6	0.003	0.05	-0.05	0.02	-0.05	-0.04	-0.06	0.010	96	96	94	95	Unlinked
Sample variance	6.8	5.5	9.6	8.6	0.005	0.003	0.001	0.001	0.002	0.002	0.001	0.001					
Mean variance	6.7	6.3	10.3	9.3													
$\overset{\wedge }{\beta_{1}}$					0.96	0.002	0.006	-0.001									
p-value					0.96	0.96	0.92	0.93									

## A Data Example

**Figure 2:**
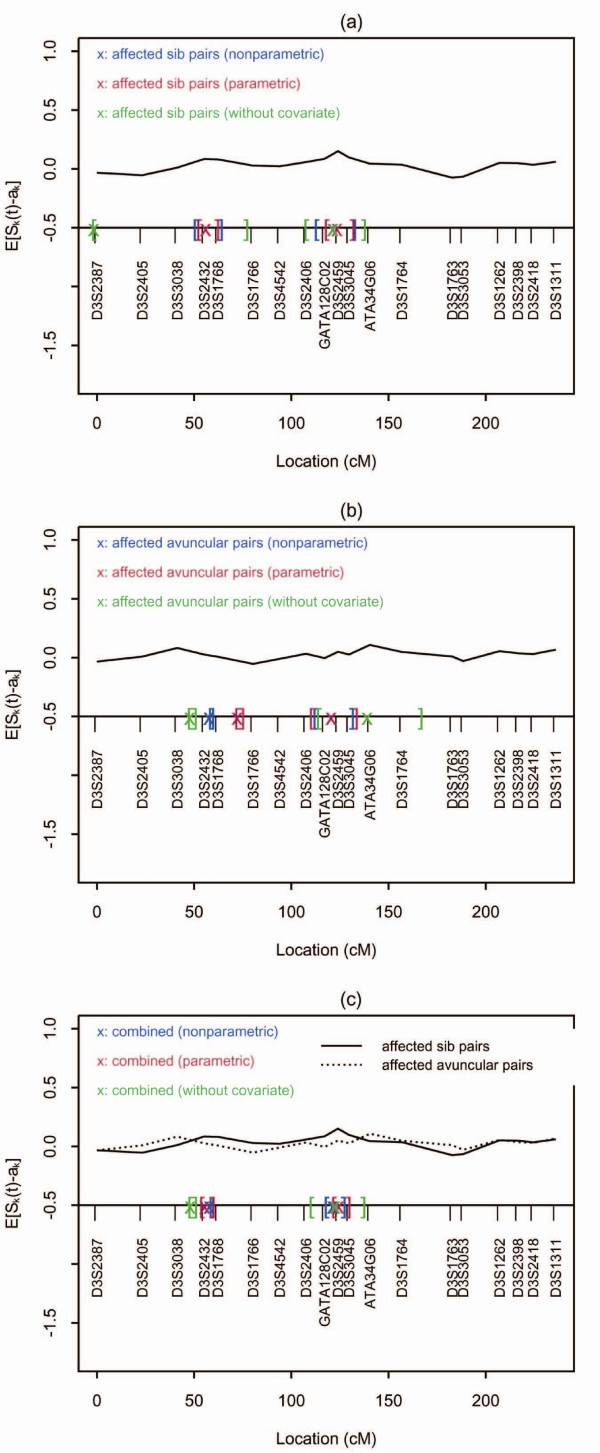
**Comparisons of estimates (denoted by "x") and their 95% CIs (denoted by brackets) for disease loci from nonparametric, parametric and without-a-covariate approaches using affected sib pairs**.

We conducted an autosome-wide scan for affected relative pairs from the COGA data [23]. Note that the disease was defined as "having psychological problems from drinking." There are 149 affected sib pairs, 8 half sib pairs, 16 first-cousins pairs, 7 grandparent-grandchild pairs, and 71 avuncular pairs in this data set. Due to the limited sample sizes for some relative pairs, we examined the linkage peak on chromosome 1 using 149 affected sib pairs and 71 avuncular pairs, with and without incorporating the quantitative covariate "Maximum number of drinks in a 24 hour period." Using both ASPs and AGPs, the disease locus was estimated to be at 113.7 cM on chromosome 1 with a 95% CI: 109.5-118.0 cM. The estimate for C_ASP _was 0.18 with a 95% CI from 0.10-0.26 (p-value = 7.6e-6), whereas the estimate for C_AAP _was 0.064 with a 95% CI from -0.0001-0.13 (p = 0.051) (Table 3 and Additional file 6, Figure S1). We also applied single locus with covariate linkage mapping using ARP to locate the disease locus and assess the significance of its covariates. The disease locus estimate was 110.8 (standard error (SE) = 1.5) and 109.2 (SE = 2.3) cM in the parametric and non-parametric approaches, respectively, using all ARPs. The p-values of the covariate in the parametric approach are 0.52 and 0.20 for ASP and AAP, respectively (Table 3). To identify a region harboring two disease loci, we plotted the empirical IBD sharing of all autosomes for ASP (because the data set included mostly sib pairs). After visually reviewing all the empirical IBD sharing on autosomes, we selected chromosome 3 as a region to illustrate our approach, as there appeared to be two disease-susceptibility loci harbored within this region (Figure 2). First, we conducted the two-locus search without incorporating the covariate (Table 4) and compared the estimates to those that did incorporate covariates. The quantitative measure "maximum number of drinks in a 24-hour period" [24] was incorporated into the linkage mapping, both parametrically (Table 5) and non-parametrically (Table 6). The 95% confidence intervals (CIs) for C or *λ *were constructed with the bootstrap re-sampling approach. A total of 1,000 replicates were obtained by re-sampling. The disease loci estimates were computed for each sample and ranked. The lower and upper limits of the 95% confidence interval were the 2.5% and 97.5% percentiles of the 1,000 replicates, respectively.

**Table 3:** One-locus search on chromosome 1 with or without incorporation of "Maximum number of drinks in a 24 hour period"

	ESTIMATE [95% CI] or (S.E.)								
	***τ *(cM)**			**C**			***λ***		
	
	**Without a covariate**	**Parametric**	**Nonparametric**	**Without a covariate**	**Parametric**	**Nonparametric**	**Without a covariate**	**Parametric**	**Nonparametric**

Using one ARP only:									
Full siblings	112.9	112.8	110.4	0.18	0.16	0.14	1.75	1.65	1.47
	(6.1)	(6.5)	(7.2)	[0.04, 0.32]	[0.001,0.32]	[0.06,0.23]	[1.14,2.84]	[1.00,2.77]	[1.13,1.87]

p-value for the covariate					0.58				

Avuncular pairs	98.8	105.0	102.8	0.08	0.20	0.036	1.46	1.74	1.37
	(12.4)	(7.8)	(6.2)	[-0.07, 0.24]	[-0.06,0.28]	[-0.10,0.23]	[0.81,2.50]	[0.79,3.56]	[0.67,2.67]

p-value for the covariate					0.23				

Using both ARPs:									
Full siblings				0.18	0.17	0.14	1.70	1.61	1.44
				[0.10, 0.26]	[0.009,0.32]	[0.045,0.23]	[1.08,2.74]	[1.02,2.70]	[1.10,1.87]

p-value for the covariate					0.52				

Avuncular pairs				0.064	0.10	0.034	1.66	1.77	1.28
				[-0.0001, 0.13]	[-0.04,0.28]	[-0.07,0.18]	[0.69,3.72]	[0.85,3.55]	[0.74,2.09]

p-value for the covariate					0.20				

Common *τ*	113.7	110.8	109.2						
	(2.2)	(1.5)	(2.3)						

**Table 4:** Simultaneous two-locus search without incorporating a covariate

	ESTIMATE (S.E.) or [95% CI]					
	*τ*_1 _(cM)	*τ*_2 _(cM)	C_1_	C_2_	*λ*_1_	*λ*_2_
Using one ARP only:						
Full siblings	1.38	124.27	-0.04	0.12	1.25	1.44
	(39.44)	(7.64)	[-0.17,0.10]	[-0.008,0.25]	[0.83,1.89]	[0.91,2.13]
Avuncular pairs	50.77	142.05	0.11	0.097	1.17	1.36
	(0.73)	(13.42)	[-0.07,0.29]	[-0.03,0.22]	[0.80,1.69]	[0.83,1.95]

Using both ARPs:						
Full siblings			0.06	0.12	1.17	1.40
			[-0.07,0.18]	[-0.01,0.24]	[0.86,1.70]	[0.90,2.08]
Avuncular pairs			0.11	0.060	1.30	1.45
			[-0.07,0.29]	[-0.034,0.154]	[0.55,2.40]	[0.68,2.28]
Common *τ*	50.98	125.43				
	(0.72)	(6.84)				

**Table 5:** Simultaneous two-locus search with incorporation of "Maximum number of drinks in a 24 hour period" - parametric approach

	ESTIMATE (S.E.) or [95% CI]					
	***τ*_1 _(cM)**	***τ*_2 _(cM)**	**C_1_**	**C_2_**	***λ*_1_**	***λ*_2_**

Using one ARP only:						
Full siblings	58.95	126.55	0.16	0.24	1.45	1.90
	(2.42)	(3.43)	[-0.01,0.32]	[0.07,0.43]	[0.91,2.76]	[1.11,7.18]
Avuncular pairs	75.14	123.55	-0.07	0.04	0.76	1.17
	(0.72)	(5.85)	[-0.15,0.21]	[-0.15,0.24]	[0.54,2.40]	[0.65,2.07]

Using both ARPs:						
Full siblings			0.16	0.23	1.46	1.82
			[-0.08,0.32]	[0.02,0.43]	[0.86,2.70]	[1.03,6.68]
Avuncular pairs			0.005	0.04	1.02	1.16
			[-0.15,0.20]	[-0.21,0.23]	[0.54,2.38]	[0.40,2.65]
Common *τ*	58.53	127.41				
	(1.47)	(1.99)				

**Table 6:** Simultaneous two-locus search with incorporation of "Maximum number of drinks in a 24 hour period" -- nonparametric approach

	ESTIMATE (S.E.) or [95% CI]					
	***τ*_1 _(cM)**	***τ*_2 _(cM)**	**C_1_**	**C_2_**	***λ*_1_**	***λ*_2_**

Using one ARP only:						
Full siblings	58.97	124.42	0.084	0.16	1.20	1.45
				[-0.004,0.27]	[0.94,1.71]	[0.99,2.21]
	(3.37)	(4.99)	[-0.03,0.21]			
Avuncular pairs	60.66	123.46	0.018	0.048	1.07	1.21
				[-0.081,0.19]	[0.64,1.58]	[0.77,1.69]
	(0.24)	(4.84)	[-0.11,0.11]			

Using both ARPs:						
Full siblings			0.083	0.16	1.20	1.45
			[-0.005,0.22]	[0.011,0.26]	[0.99,1.79]	[1.02,2.09]
Avuncular pairs			0.017	0.051	1.07	1.23
			[-0.11,0.12]	[-0.052,0.18]	[0.63,1.62]	[0.81,2.10]
Common *τ*	60.81	124.24				
	(0.16)	(2.29)				

The standard errors for the estimates of the disease loci were always smaller when using the entire data set with both sibpairs and avuncular pairs, compared to the estimates using sib pairs or avuncular pairs alone. Compared to the approach without the covariate, the relative efficiencies (each defined as the ratio of reversed variance estimates for the disease locus estimates) in estimating *τ*_1 _and *τ*_2 _are 20.25 ((0.7/0.2)^2^) and 8.92 ((6.84/2.29)^2^) for the non-parametric approach (Table 6) and 0.24 ((0.72/1.47)^2^) and 11.8 ((6.84/1.99)^2^) for the parametric approach (Table 5). The average estimated *C*_1 _and *C*_2 _were 0.084 and 0.16 for affected sibpairs in the non-parametric approach (Table 6), and were 0.16 and 0.24 in the parametric approach (Table 5). The corresponding risk ratios *λ*_*l *_for these two loci in sib pairs within the non-parametric approach were 1.20 (95% CI: 0.99 to 1.79) and 1.45 (95% CI: 1.02 to 2.09), respectively (Table 6). The C value (or risk ratio) at *τ*_2 _(0.237, 95% CI: 0.066 to 0.430) was higher than that at *τ*_1 _(0.156, 95% CI: -0.014 to 0.319), and it was marginally significant after incorporation of the covariate (Table 5). The *C*_*l *_and *λ*_*l *_values estimated from avuncular pairs were smaller than those estimated from sib pairs (Tables 4, 5, 6) with incorporation of the covariate; however, this difference was not statistically significant. Since there was no evidence of linkage at *τ*_1_, the estimate for *τ*_1 _varied in the three approaches.

## Discussion and Conclusions

Many complex diseases involve multiple loci as well as multiple quantitative biological markers or quantitative risk factors. Incorporating covariates into linkage analysis is not only helpful for the identification of disease loci but is also informative with respect to disease etiology. In family-based studies, data are often available for larger pedigrees with multiple relative pairs, and therefore it is desirable to have linkage mapping approaches that can use these potentially informative data. In addition, different types of ARPs may have the potential of providing some insight into the underlying genetic mechanism [19]. Applying a one-locus model to localize a disease gene when there are actually two linked disease genes in the region is likely to estimate the two true disease gene locations inaccurately, while the corresponding effect size tends to be over-estimated [20]. Therefore, we extended a robust multipoint linkage approach in simultaneously mapping two linked disease loci while using affected relative pairs with an incorporation of quantitative covariates. A series of intensive simulation studies were conducted to examine the performance of the approach when the incorporated covariate was a quantitative trait under a variety of genetic models or when the trait was a risk factor associated with a disease locus. The simulation study suggested that incorporating a quantitative covariate, which also happened to be a quantitative trait, helped improve the efficiency of the disease-locus estimate, regardless of the genetic models that actually underlie the incorporated covariate. It seems that the underlying genetic models of the quantitative covariate (trait) did not have much impact on the efficiency in estimating *τ*_*l*_, *l *= 1,2. In addition, the inclusion of different relative pairs would make the sample size larger and improve the efficiency of the disease-locus localization when the different relative pairs share common disease loci; this would be particularly true when the genetic effect of the disease loci is small or modest. When the covariate was directly related to the liability of the disease, the efficiency improvement was greater than when it was not directly related to the disease liability; when the covariate was associated with only one disease locus, incorporating the covariate helped improve the efficiency of that locus' estimate more than that of the other locus. The position of the QTL for a quantitative trait (as a covariate) might slightly affect the accuracy of the disease-loci localization; the accuracy was similar to the situation in which no covariates were incorporated given an unlinked relationship between the QTL and disease locus. Investigators can choose to incorporate covariates that improve efficiency in disease-loci estimation. Our example of an alcoholism study illustrates that incorporating a quantitative covariate into the linkage mapping helps improve the efficiency of disease-loci estimates in the two-locus models by either the parametric approach or the nonparametric approach. The assessment of associations between the disease loci and covariates helps resolve the underlying genetic mechanism of the disease. Using all affected relative pairs to estimate the common disease loci could also enhance the efficiency in estimating disease loci, and, furthermore, it could help dissect disease etiology by assessing risk ratios among different types of relative pairs.

Although the proposed approaches can be quite helpful and can also be widely applied to localize disease loci for complex diseases, they are built upon the assumption of a two-locus disease mechanism. Bias may arise when a region harboring one locus only or more than two linked loci is examined. In addition, since the relationships between the genetic effects on the two disease loci and covariates are modeled separately, the number of parameters may easily be increased when (1) several covariates are incorporated simultaneously; or (2) regression relationships between the genetic effects on the two disease loci and covariates are not assumed to be identical; or (3) several relative types are analyzed. Additionally, since fitting an incorrect model can lead to biased estimates with anti-conservative confidence intervals, it is important to decide whether a one-locus or two-locus model is more appropriate. In practice, it is always helpful to check the empirical plot (as shown in Figure 2) to determine how many "peaks" are present in the region of interest. If there is only one "peak," a one-locus model might be more appropriate than a two-locus model. If more than two peaks are present, it might be helpful to split the region into multiple smaller regions containing only two peaks each. Indeed, it is always helpful to apply both one-locus and two-locus models and evaluate which model fits the data better. In addition, the test developed by Biernacka et al. [21] can be used to help choose an appropriate model.

The proposed approaches allow gene-gene and gene-environment interactions to be assessed. As complex diseases often involve more than two disease genes, further efforts to extend this method to situations involving more than two genes are warranted. In addition, as the regions identified through linkage mapping are quite wide and may harbor numerous genes, future approaches should be developed to identify potential causal polymorphisms by the joint modeling of linkage and association.

## Authors' contributions

YFC, JMC and KYL have made contributions to the theory derivation, simulation study, statistical modeling and draft of the manuscript. CYL participated in the design of the study and performed the simulation studies and data analysis. All authors read and approved the final manuscript.

## Supplementary Material

Additional file 1 **Table S1**. Expected alleles shared IBD at location t for five types of ARPs and functions relating *λ *to C Click here for file

Additional file 2 **Appendix**. Theoretical derivations. Click here for file

Additional file 3 **Table S2**. The two-locus genetic model used in simulation studies. Click here for file

Additional file 4 **Tables S3-S5**. Table S3. Comparisons of estimate for *τ *with and without incorporation of a quantitative covariate under one-locus recessive model (a). Table S4. Comparisons of estimate for *τ *with and without incorporation of a quantitative covariate under one-locus dominant model (b). Table S5. Comparisons of estimate for *τ *with and without incorporation of a quantitative covariate under one-locus additive model (c). Click here for file

Additional file 5 **Table S6**. Simultaneous two-locus search without incorporating a covariate. Click here for file

Additional file 6 **Figure S1**. Comparisons of estimates (denoted by "x") and their 95% CIs (denoted by brackets) for the disease locus on chromosome one from nonparametric, parametric and without-a-covariate approaches using affected sib pairs Click here for file
