# Supplementary material for: Incorporation of covariates in simultaneous localization of two linked loci using affected relative pairs
Source: BMC Genet. 2010 Jul 14;11:67. doi: 10.1186/1471-2156-11-67 (PMC3247820; doi:10.1186/1471-2156-11-67)
Supplement: Additional file 1 — Table S1. Expected alleles shared IBD at location t for five types of ARPs and functions relating λ to C [file 1471-2156-11-67-S1.DOC]

Table S1. Expected alleles shared IBD at location *t* for five types of ARPs and functions relating $\lambda$ to C

| **ARP** | **a_k_** | **b_k_(d)** | **C($\lambda$)** | $\lambda$**(C)** |
| --- | --- | --- | --- | --- |
| **SP** | 1 | exp(-0.04d) | $\frac{\lambda-1}{2\lambda}$ | $\frac{1}{1-2C}$ |
| **HS** | 1/2 | exp(-0.04d) | $\frac{\lambda-1}{2(\lambda+1)}$ | $\frac{1+2C}{1-2C}$ |
| **FC** | 1/4 | 1/2*exp*(–0.04*d*) + 1/3*exp*(–0.06*d*) + 1/6*exp*(–0.08*d*) | $\frac{3(\lambda-1)}{4(\lambda+3)}$ | $\frac{12C+3}{3-4C}$ |
| **GP** | 1/2 | *exp*(–0.02*d*) | $\frac{\lambda-1}{2(\lambda+1)}$ | $\frac{1+2C}{1-2C}$ |
| **AP** | 1/2 | 1/2*exp*(–0.04*d*) + 1/2*exp*(–0.06*d*) | $\frac{\lambda-1}{2(\lambda+1)}$ | $\frac{1+2C}{1-2C}$ |
