# Supplementary material for: Incorporation of covariates in simultaneous localization of two linked loci using affected relative pairs
Source: BMC Genet. 2010 Jul 14;11:67. doi: 10.1186/1471-2156-11-67 (PMC3247820; doi:10.1186/1471-2156-11-67)
Supplement: Additional file 2 — Appendix. Theoretical derivations. [file 1471-2156-11-67-S2.DOC]

**Appendix**

We argue that the estimate, $\hat{\tau}_{l}$, $l=1,2$ obtained by solving the generalized estimating equation (1), asymptotically follows a normal distribution with mean $\tau_{l}$ and variance $\Sigma^{-1}$ as described in the Methods section.

Assume the relative pairs have the same disease loci. Let $\delta=(\tau_{1},\tau_{2})$,$C(\delta)=(C_{1k}(\tau_{1}),C_{2k}(\tau_{2}))$, and define the quasi-score vector

$\begin{matrix} \text{ }U_{ki}(\delta;C(\delta)) \\ =\left(\partial\mu_{ki}(\delta)/\partial\delta\right)^{'}Cov^{-1}(S_{ki})(S_{ki}-\mu_{ki}(\delta)). \end{matrix}$ (5)

Since $\partial\mu_{ki}(\delta)/\partial\tau_{1}\partial\tau_{2}=0$, the quasi-score vector can be written w.r.t. $\tau_{1}$ and $\tau_{2}$ separately as,

$\begin{matrix} \text{ }U_{lki}(\tau_{l};C_{lk}(\tau_{l})) \\ =\left(\partial\mu_{ki}(\tau_{l})/\partial\tau_{l}\right)^{'}Cov^{-1}(S_{ki})(S_{ki}-\mu_{ki}(\tau_{l})), \end{matrix}$ (6)

and the quasi-information matrix

$I_{lki}(\tau_{l};C_{lk}(\tau_{l}))=-\partial U_{lki}(\tau_{l};C_{lk}(\tau_{l}))/\partial\tau_{l}$, (7)

for $l=1,2$, where $S_{ki}$ and $\mu_{ki}(\tau_{l})$ are defined in (1), and $\tau_{1}$ and $\tau_{2}$ are the true locations of trait loci. Using these notations and under regularity conditions, the estimate $\hat{\tau}_{l}$ is obtained by solving the estimating equation

, (8)

which follows an asymptotic normal distribution with mean $\tau_{l}$ and variance $I_{lk}(\tau_{l};C_{lk}(\tau_{l}))^{-1}$, where

$I_{lk}(\tau_{l};C_{lk}(\tau_{l}))=\sum_{i=1}^{n_{k}} I_{lki}(\tau_{l};C_{lk}(\tau_{l}))$. (9)

In practice, the function $C_{lk}$ is unknown, and it is replaced with the estimate $\hat{C}_{lk}$ , obtained by the proposed parametric and non-parametric methods and which has the consistency properties that follow: $|\hat{C}_{lk}-C_{lk}|=O_{p}(\sigma_{n_{k}})$, for a sequence of $\sigma_{n_{k}}\to0$, satisfying $\sigma_{n_{k}}=O\left(1/\sqrt{n_{k}}\right)$. In the non-parametric approach, this depends on the particular smoother and the choice of smoothing parameters. Let be the estimate of $E(S(\tau_{l}))$ with $\bar{\tau}_{l}$ lying on the line segment joining $\tau_{l}$ and $\hat{\tau}_{l}$. By applying the Taylor expansion to the estimating equation , it follows that

$\begin{matrix} \text{ }\sqrt{n_{k}}(\hat{\tau}_{l}-\tau_{l})) \\ =\left\{-\frac{1}{n_{k}}I_{l}(\bar{\tau}_{l};\hat{C}_{lk}(\hat{\tau}_{l}))\right\}^{-1}\times\left\{\frac{1}{\sqrt{n_{k}}}U_{lk}(\tau_{l};\hat{C}_{lk}(\hat{\tau}_{l}))\right\}. \end{matrix}$ (10)

Thus, there exists a $\hat{\tau}_{l}$ satisfying $\hat{\tau}_{l}-\tau_{l}=O_{p}\left(1/\sqrt{n_{k}}\right)$. It can then be shown that the first term on the right-hand-side is $O_{p}(1)$ and the second term is $\frac{1}{\sqrt{n_{k}}}U_{l}(\tau_{l};C_{lk}(\tau_{l}))+o_{p}(1)$. By Slutsky’s theorem and the above assertion of the asymptotic normality, the asymptotic property of $\hat{\tau}_{l}$ is established accordingly. Since the covariance $Cov(S_{ki})$ is unknown and can only be approximated, the robust sandwich-type variance estimate for $\hat{\tau}_{l}$ is suggested to account for the within-subject correlations [17].

For relative type $k$, the variance estimate of $\hat{\tau}_{lk}=\hat{\mathrm{v}}\mathrm{ar}(\hat{\tau}_{lk})=\hat{\Sigma}_{lk1}^{-1}\hat{\Sigma}_{lk2}\hat{\Sigma}_{lk1}^{-1}$,

where

$$\hat{\Sigma}_{lk1}\text{ }=\text{ }\left.\sum_{i=1}^{n_{ki}} \left(\frac{\partial\mu_{ki}(\tau_{l})}{\partial\tau_{l}}\right)\text{ }Cov^{-1}(S_{ki})\text{ }\left(\frac{\partial\mu_{ki}(\tau_{l})}{\partial\tau_{l}}\right)^{'}\right|_{\tau_{l}=\hat{\tau}_{l}}\text{,}$$

$$\begin{matrix} \text{and }\hat{\Sigma}_{lk2}\text{ }=\text{ }\sum_{i=1}^{n_{ki}} \left(\frac{\partial\mu_{ki}(\tau_{l})}{\partial\tau_{l}}\right)^{'}Cov^{-1}(S_{ki})\text{ }(S_{ki}-\mu_{ki}(\tau_{l})) \\ \text{ }\times\text{ }\left.(S_{ki}-\mu_{ki}(\tau_{l}))'\text{ }Cov^{-1}(S_{ki})\text{ }\left(\frac{\partial\mu_{ki}(\tau_{l})}{\partial\tau_{l}}\right)\text{ }\right|_{\tau_{l}=\hat{\tau}_{l}}. \end{matrix}$$

Similarly, $\delta$ and variance of $\hat{\delta}$ can be estimated by combining all types of relative pairs if the assumption of common $\delta$ is made, by the variance estimate of ,

where

$\hat{\Sigma}_{l1}\text{ }=\text{ }\left.\sum_{k=1}^{K} \sum_{i=1}^{n_{ki}} \left(\frac{\partial\mu_{ki}(\tau_{l})}{\partial\tau_{l}}\right)\text{ }Cov^{-1}(S_{ki})\text{ }\left(\frac{\partial\mu_{ki}(\tau_{l})}{\partial\tau_{l}}\right)^{'}\right|_{\tau_{l}=\hat{\tau}_{l}}\text{,}$ (11)

$$\begin{matrix} \text{and }\hat{\Sigma}_{l2}\text{ }=\text{ }\sum_{k=1}^{K} \sum_{i=1}^{n_{ki}} \left(\frac{\partial\mu_{ki}(\tau_{l})}{\partial\tau_{l}}\right)^{'}Cov^{-1}(S_{ki})\text{ }(S_{ki}-\mu_{ki}(\tau_{l})) \\ \text{ }\times\text{ }\left.(S_{ki}-\mu_{ki}(\tau_{l}))'\text{ }Cov^{-1}(S_{ki})\text{ }\left(\frac{\partial\mu_{ki}(\tau_{l})}{\partial\tau_{l}}\right)\text{ }\right|_{\tau_{l}=\hat{\tau}_{l}}\text{ . (12)} \end{matrix}$$
