# Supplementary material for: Incorporation of covariates in simultaneous localization of two linked loci using affected relative pairs
Source: BMC Genet. 2010 Jul 14;11:67. doi: 10.1186/1471-2156-11-67 (PMC3247820; doi:10.1186/1471-2156-11-67)
Supplement: Additional file 3 — Table S2. The two-locus genetic model used in simulation studies. [file 1471-2156-11-67-S3.DOC]

Table S2. The two-locus genetic model used in simulation studies

| Model | Penetrance matrix | | | | Allele frequencies | Prevalence | τ_2_-τ_1_ | C_11_, C_21_  C_14_, C_24_ |
| --- | --- | --- | --- | --- | --- | --- | --- | --- |
| Model B in Biernacka et. al. (2005) | | aa | aA | AA |  |  |  |  |
| A, a: alleles for locus 1 | bb | 0.01 | 0.01 | 0.80 | Pr(A)=0.2 | Approx 7% | 40 cM | 0.34,0.34 |
| B, b: alleles for locus 2 | bB | 0.01 | 0.01 | 0.80 | Pr(B)=0.2 |  |  | 0.19,0.12 |
|  | BB | 0.80 | 0.80 | 0.80 |  |  |  |  |

C_11_, C_21_: genetic effects atτ_1_ and τ_2_ for affected sib pairs (relative pair type = 1) respectively

C_14_, C_24_: genetic effects atτ_1_ and τ_2_ for affected grandparent-grandchild pairs (relative pair type = 4) respectively
