# Supplementary material for: Incorporation of covariates in simultaneous localization of two linked loci using affected relative pairs
Source: BMC Genet. 2010 Jul 14;11:67. doi: 10.1186/1471-2156-11-67 (PMC3247820; doi:10.1186/1471-2156-11-67)
Supplement: Additional file 4 — Tables S3-S5. Table S3. Comparisons of estimate for τ with and without incorporation of a quantitative covariate under one-locus recessive model (a). Table S4. Comparisons of estimate for τ with and without incorporation of a quantitative covariate under one-locus dominant model (b). Table S5. Comparisons of estimate for τ with and without incorporation of a quantitative covariate under one-locus additive model (c). [file 1471-2156-11-67-S4.DOC]

Table S3. Comparisons of estimate for$\tau$with and without incorporation of a quantitative covariate under one-locus recessive model (a)

Non-parametric (i) Pleiotropy (ii) Co-incident (iii) Unlinked (iv) Age at onset

| Estimate | ASP_GF* |  | ASP | AGP^§^ | COM^†^ |  | ASP | AGP | COM |  | ASP | AGP | COM |  | ASP | AGP | COM |
| --- | --- | --- | --- | --- | --- | --- | --- | --- | --- | --- | --- | --- | --- | --- | --- | --- | --- |
| Bias | 0.019 |  | 0.054 | 0.039 | 0.038 |  | 0.068 | 0.13 | 0.070 |  | 0.027 | -0.024 | 0.0077 |  | 0.030 | -0.058 | 0.021 |
| Sample Variance | 3.32 |  | 2.79 | 14.41 | 2.63 |  | 2.64 | 14.64 | 2.53 |  | 2.88 | 14.77 | 2.73 |  | 2.27 | 12.73 | 2.18 |
| Mean Variance | 2.88 |  | 2.36 | 10.59 | 2.21 |  | 2.39 | 10.21 | 2.20 |  | 2.51 | 10.98 | 2.32 |  | 1.92 | 8.93 | 1.81 |
| 95% Coverage Probability | 0.96 |  | 0.92 | 0.90 | 0.92 |  | 0.93 | 0.90 | 0.93 |  | 0.93 | 0.90 | 0.92 |  | 0.92 | 0.90 | 0.92 |

*ASP_GF: Using Affected sib pairs without incorporating a covariate by GeneFinder.

^§^AGP: Affected grandparent-grandchild pairs

^†^COM: Combine ASP and AGP

Parametric (i) Pleiotropy (ii) Co-incident (iii) Unlinked (iv) Age at onset

| Estimate | ASP_GF |  | ASP | AGP | COM |  | ASP | AGP | COM |  | ASP | AGP | COM |  | ASP | AGP | COM |
| --- | --- | --- | --- | --- | --- | --- | --- | --- | --- | --- | --- | --- | --- | --- | --- | --- | --- |
| Bias | 0.019 |  | -0.045 | 0.019 | 0.054 |  | 0.019 | 0.0019 | 0.013 |  | 0.024 | 0.065 | 0.020 |  | -0.0075 | -0.075 | -0.11 |
| Sample Variance | 3.32 |  | 3.90 | 11.04 | 4.22 |  | 1.20 | 11.76 | 1.23 |  | 1.20 | 11.34 | 1.23 |  | 4.58 | 10.65 | 9.77 |
| Mean Variance | 2.88 |  | 1.45 | 8.75 | 1.12 |  | 1.45 | 9.09 | 1.12 |  | 1.45 | 8.87 | 1.12 |  | 1.35 | 7.97 | 1.06 |
| 95% Coverage Probability | 0.96 |  | 0.97 | 0.92 | 0.93 |  | 0.97 | 0.92 | 0.94 |  | 0.97 | 0.91 | 0.95 |  | 0.93 | 0.91 | 0.88 |

Table S4. Comparisons of estimate for$\tau$with and without incorporation of a quantitative covariate under one-locus dominant model (b)

Non-parametric (i) Pleiotropy (ii) Co-incident (iii) Unlinked (iv) Age at onset

| Estimate | ASP_GF |  | ASP | AGP | COM |  | ASP | AGP | COM |  | ASP | AGP | COM |  | ASP | AGP | COM |
| --- | --- | --- | --- | --- | --- | --- | --- | --- | --- | --- | --- | --- | --- | --- | --- | --- | --- |
| Bias | -0.050 |  | -0.038 | 0.040 | 0.071 |  | 0.23 | 0.30 | 0.22 |  | -0.058 | 0.035 | -0.017 |  | 0.045 | 0.10 | 0.044 |
| Sample Variance | 12.30 |  | 9.32 | 15.66 | 6.48 |  | 8.04 | 16.37 | 6.04 |  | 8.96 | 16.85 | 6.58 |  | 8.46 | 16.12 | 5.91 |
| Mean Variance | 8.78 |  | 6.63 | 12.08 | 4.88 |  | 6.30 | 11.74 | 4.67 |  | 6.92 | 12.69 | 5.12 |  | 6.40 | 11.33 | 4.71 |
| 95% Coverage Probability | 0.89 |  | 0.91 | 0.90 | 0.91 |  | 0.91 | 0.91 | 0.90 |  | 0.92 | 0.90 | 0.92 |  | 0.90 | 0.89 | 0.92 |

Parametric (i) Pleiotropy (ii) Co-incident (iii) Unlinked (iv) Age at onset

| Estimate | ASP_GF |  | ASP | AGP | COM |  | ASP | AGP | COM |  | ASP | AGP | COM |  | ASP | AGP | COM |
| --- | --- | --- | --- | --- | --- | --- | --- | --- | --- | --- | --- | --- | --- | --- | --- | --- | --- |
| Bias | -0.050 |  | -0.030 | 0.092 | 0.037 |  | -0.033 | 0.082 | 0.0053 |  | -0.00031 | 0.085 | 0.014 |  | -0.016 | 0.12 | 0.039 |
| Sample Variance | 12.30 |  | 9.85 | 10.85 | 2.71 |  | 3.37 | 11.24 | 2.68 |  | 3.28 | 11.06 | 2.65 |  | 3.40 | 12.21 | 2.63 |
| Mean Variance | 8.78 |  | 7.40 | 9.99 | 2.20 |  | 3.68 | 10.04 | 2.21 |  | 3.65 | 10.15 | 2.20 |  | 3.61 | 9.77 | 2.17 |
| 95% Coverage Probability | 0.89 |  | 0.93 | 0.93 | 0.93 |  | 0.96 | 0.93 | 0.93 |  | 0.97 | 0.93 | 0.93 |  | 0.97 | 0.93 | 0.93 |

Table S5. Comparisons of estimate for$\tau$with and without incorporation of a quantitative covariate under one-locus additive model (c)

Non-parametric (i) Pleiotropy (ii) Co-incident (iii) Unlinked (iv) Age at onset

| Estimate | ASP_GF |  | ASP | AGP | COM |  | ASP | AGP | COM |  | ASP | AGP | COM |  | ASP | AGP | COM |
| --- | --- | --- | --- | --- | --- | --- | --- | --- | --- | --- | --- | --- | --- | --- | --- | --- | --- |
| Bias | -0.15 |  | -0.16 | 0.030 | -0.16 |  | 0.072 | 0.25 | 0.031 |  | -0.12 | -0.011 | -0.13 |  | -0.13 | 0.021 | -0.097 |
| Sample Variance | 12.90 |  | 7.00 | 18.16 | 5.72 |  | 7.69 | 19.58 | 5.70 |  | 8.09 | 19.19 | 6.00 |  | 6.17 | 17.21 | 5.10 |
| Mean Variance | 8.62 |  | 5.96 | 12.92 | 4.61 |  | 6.26 | 12.52 | 4.73 |  | 6.96 | 13.48 | 5.15 |  | 5.09 | 11.68 | 4.04 |
| 95% Coverage Probability | 0.88 |  | 0.92 | 0.89 | 0.92 |  | 0.92 | 0.88 | 0.92 |  | 0.92 | 0.89 | 0.93 |  | 0.92 | 0.88 | 0.92 |

Parametric (i) Pleiotropy (ii) Co-incident (iii) Unlinked (iv) Age at onset

| Estimate | ASP_GF |  | ASP | AGP | COM |  | ASP | AGP | COM |  | ASP | AGP | COM |  | ASP | AGP | COM |
| --- | --- | --- | --- | --- | --- | --- | --- | --- | --- | --- | --- | --- | --- | --- | --- | --- | --- |
| Bias | -0.15 |  | -0.089 | 0.078 | -0.078 |  | -0.12 | 0.036 | -0.093 |  | -0.12 | 0.033 | -0.089 |  | -0.083 | -0.010 | -0.096 |
| Sample Variance | 12.90 |  | 3.20 | 13.63 | 2.67 |  | 3.21 | 13.71 | 2.83 |  | 3.21 | 13.37 | 2.75 |  | 3.53 | 13.10 | 2.75 |
| Mean Variance | 8.62 |  | 3.61 | 10.62 | 2.20 |  | 3.64 | 10.71 | 2.21 |  | 3.64 | 10.78 | 2.21 |  | 3.59 | 10.40 | 2.18 |
| 95% Coverage Probability | 0.88 |  | 0.97 | 0.90 | 0.92 |  | 0.97 | 0.91 | 0.92 |  | 0.97 | 0.91 | 0.93 |  | 0.97 | 0.91 | 0.93 |
