# Supplementary material for: Incorporation of covariates in simultaneous localization of two linked loci using affected relative pairs
Source: BMC Genet. 2010 Jul 14;11:67. doi: 10.1186/1471-2156-11-67 (PMC3247820; doi:10.1186/1471-2156-11-67)
Supplement: Additional file 5 — Table S6. Simultaneous two-locus search without incorporating a covariate. [file 1471-2156-11-67-S5.DOC]

Table S6. Simultaneous two-locus search without incorporating a covariate

|  | Disease Loci (cM) | |  | | Genetic Effect | | | | | |  | | 95% coverage probability (%) | |  |
| --- | --- | --- | --- | --- | --- | --- | --- | --- | --- | --- | --- | --- | --- | --- | --- |
|  |  |  |  | | ASP | |  | | AGP | |  | |  |  |  |
|  | $\tau_{1}$ | $\tau_{2}$ |  | | C_11_ | C_21_ |  | | C_14_ | C_24_ |  | | $\tau_{1}$ | $\tau_{2}$ |  |
| Estimate | 34.9 | 75.1 | | 0.343 | | 0.343 | | 0.140 | | 0.139 | | 92 | | 93 | |
| Bias | -0.1 | 0.1 | | 0.006 | | 0.006 | | -0.046 | | 0.019 | |  | |  | |
| Sample variance | 10.0 | 9.2 | | 0.003 | | 0.003 | | 0.002 | | 0.002 | |  | |  | |
| Mean variance | 7.5 | 6.9 | | 0.003 | | 0.003 | | 0.00002 | | 0.00001 | |  | |  | |
| P-value |  |  | | 3.79E-10 | | 3.79E-10 | | <1.0E-15 | | <1.0E-15 | |  | |  | |
