# Supplementary figures and images for: Incorporation of covariates in simultaneous localization of two linked loci using affected relative pairs
Source: BMC Genet. 2010 Jul 14;11:67. doi: 10.1186/1471-2156-11-67 (PMC3247820; doi:10.1186/1471-2156-11-67)

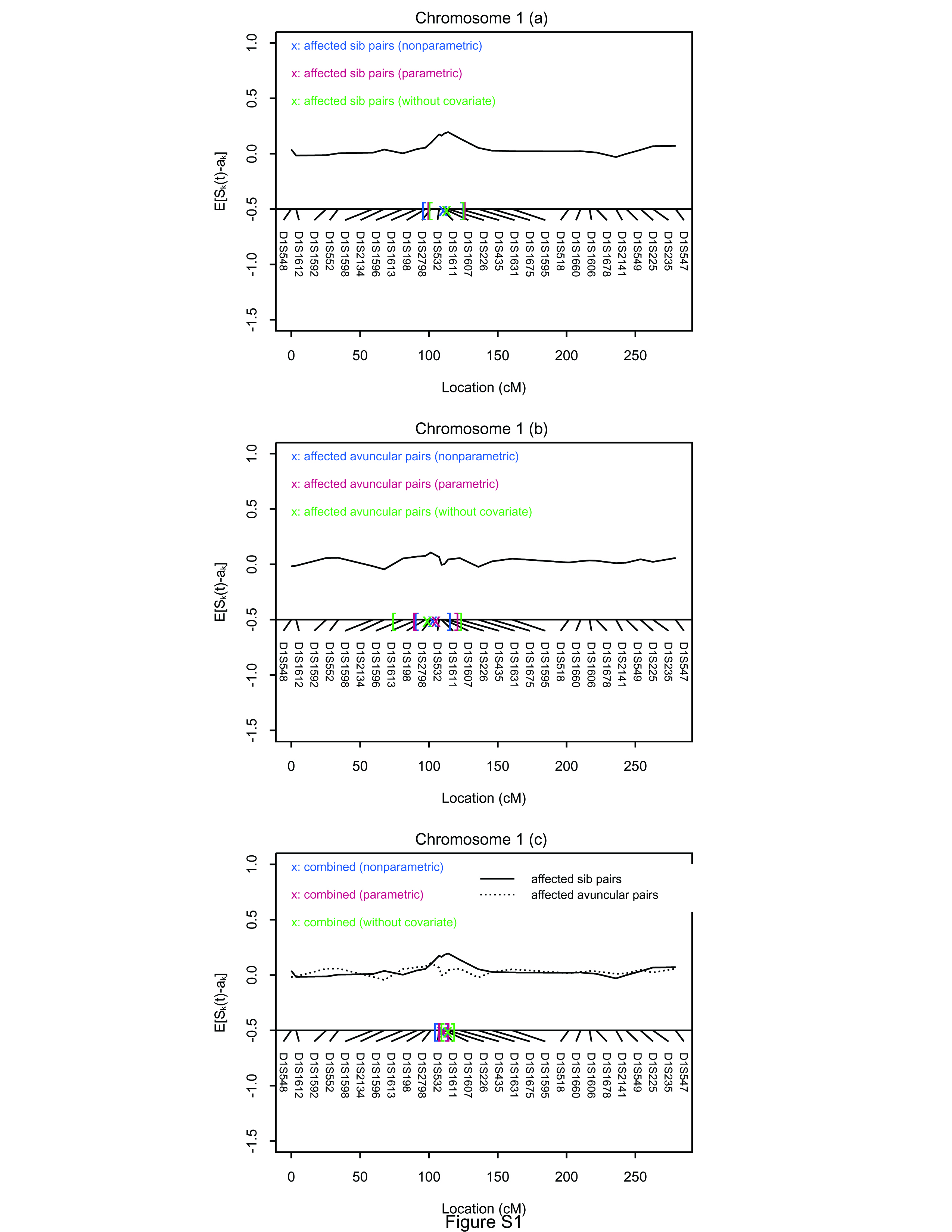

Supplement: Additional file 6 — Figure S1. Comparisons of estimates (denoted by "x") and their 95% CIs (denoted by brackets) for the disease locus on chromosome one from nonparametric, parametric and without-a-covariate approaches using affected sib pairs [file 1471-2156-11-67-S6.JPEG]
